# Hydrogen gas protects IP3Rs by reducing disulfide bridges in human keratinocytes under oxidative stress

**DOI:** 10.1038/s41598-017-03513-2

**Published:** 2017-06-15

**Authors:** Ching-Ying Wu, Wen-Li Hsu, Ming-Hsien Tsai, Jui-Lin Liang, Jian-He Lu, Chia-Jung Yen, Hsin-Su Yu, Mami Noda, Chi-Yu Lu, Chu-Huang Chen, Shian-Jang Yan, Tohru Yoshioka

**Affiliations:** 1 Department of Dermatology, Kaohsiung Municipal Ta-Tung Hospital, Kaohsiung Medical University Hospital, Kaohsiung Medical University, No. 68, Jhonghua 3rd Rd, Cianjin District, Kaohsiung, 80145 Taiwan; 2 The Institute of Basic Medical Sciences, College of Medicine, National Cheng Kung University, 1 University Road, Tainan, 70101 Taiwan; 3 Center for Lipid Biosciences, Kaohsiung Medical University Hospital, Kaohsiung Medical University, No. 100, Shih-Chuan 1st Road, Kaohsiung, 80708 Taiwan; 4 Chi Mei Medical Center, No. 201, Taikang, Taikang Vil., Liuying Dist., Tainan City, 736 Taiwan; 5 Graduate Institute of Medicine, School of Medicine, Kaohsiung Medical University, No. 100, Shih-Chuan 1st Road, Kaohsiung, 80708 Taiwan; 6 Department of Dermatology, Kaohsiung Medical University, No. 100, Shih-Chuan 1st Road, Kaohsiung, 80708 Taiwan; 7 National Health Research Institutes, Distinguished Investigator, National Environmental Health Research Center, No. 35, Keyan Road, Zhunan Town, Miaoli County, 35053 Taiwan; 8 Laboratory of Pathophysiology, Graduate School of Pharmaceutical Sciences, Kyushu University, 3-1-1Maidashi, Higashi-ku, Fukuoka, 812-8581 Japan; 9 Department of Biochemistry, College of Medicine, Kaohsiung Medical University, 100, Shih-Chuan 1st Road, Kaohsiung, 80708 Taiwan; 10 Lipid Science and Aging Research Center, Kaohsiung Medical University, No. 100, Shih-Chuan 1st Road, Kaohsiung, 80708 Taiwan; 11 Vascular and Medicinal Research, Texas Heart Institute, Houston, TX USA; 12 New York Heart Research Foundation, Mineola, NY USA; 13 Department of Physiology, College of Medicine, National Cheng Kung University, 1 University Road, Tainan, 70101 Taiwan

## Abstract

Based on the oxidative stress theory, aging derives from the accumulation of oxidized proteins induced by reactive oxygen species (ROS) in the cytoplasm. Hydrogen peroxide (H_2_O_2_) elicits ROS that induces skin aging through oxidation of proteins, forming disulfide bridges with cysteine or methionine sulfhydryl groups. Decreased Ca^2+^ signaling is observed in aged cells, probably secondary to the formation of disulfide bonds among Ca^2+^ signaling-related proteins. Skin aging processes are modeled by treating keratinocytes with H_2_O_2_. In the present study, H_2_O_2_ dose-dependently impaired the adenosine triphosphate (ATP)-induced Ca^2+^ response, which was partially protected via co-treatment with β-mercaptoethanol, resulting in reduced disulfide bond formation in inositol 1, 4, 5-trisphosphate receptors (IP_3_Rs). Molecular hydrogen (H_2_) was found to be more effectively protected H_2_O_2_-induced IP_3_R1 dysfunction by reducing disulfide bonds, rather than quenching ROS. In conclusion, skin aging processes may involve ROS-induced protein dysfunction due to disulfide bond formation, and H_2_ can protect oxidation of this process.

## Introduction

The oxidative stress theory of aging suggests that aging results from the accumulation of aberrant proteins in the cytosol, chemical damage to macromolecules, and mitochondrial DNA changes^1^. This theory began as a proposal that oxygen was poisonous^2^, followed by the notion that reactive oxygen species (ROS) are a cause of aging, and was eventually modified as the oxidative stress theory in 1972^3^. Among the large number of aging models proposed to date, the oxidative stress hypothesis is considered the most likely, because ROS are continuously produced in aerobic cells. Stepwise reduction of O_2_ produces several ROS, such as superoxide radicals $(\cdot {{\rm{O}}}_{2}^{-})$, hydrogen peroxide (H_2_O_2_), and hydroxyl radicals $(\cdot {\rm{HO}})$. ROS-induced damage of many types of cellular components is supported by a plethora of cellular and biologic data from various model systems and organisms^4^. Despite the enormous amount of data, however, the molecular mechanisms of aging are not clearly elucidated.

H_2_O_2_ is generally used as an instrumental ROS species despite some limitations, such as the complex effects of H_2_O_2_ on catalase. Further, H_2_O_2_ may be transported across the membrane by aquaporin channels^5^ and act as superoxide anions, major ROS released from the mitochondria that are converted to H_2_O_2_ by superoxide dismutase^6^, and the increased release of H_2_O_2_ mimics the aging process^7^. Interestingly, H_2_O_2_ selectively allows for the oxidization of cysteine or methionine sulfhydryl groups to sulfenic acid or disulfide bonds^8, 9^, inducing cytoplasm protein dysfunction with the formation of disulfide bonds^10^. Therefore, H_2_O_2_ impairs various physiologic processes via the oxidation of thiols, especially those in proteins. In addition, over the last 20 years, Ca^2+^ signaling has been identified as crucial for normal physiologic processes^11–13^. The effect of protein oxidation (or effect of aging) on Ca^2+^ signaling is therefore an important topic. Based on several published papers, most aged cells have decreased Ca^2+^ responses in the endoplasmic reticulum and decreased Ca^2+^ release^14–16^. These findings suggest that inositol 1, 4, 5-trisphosphate receptors (IP_3_Rs)-mediated Ca^2+^ release from ER must also be decreased^14^. Accordingly, the aging-related reduction of Ca^2+^ signaling may be mimicked by H_2_O_2_-induced disulfide bond formation.

Recently, a large number of studies have demonstrated that H_2_ gas selectively reduces ROS, especially hydroxyl radicals, and can strongly slow the rate of aging processes or the progression of aging-related diseases, such as ischemia, reperfusion brain injury, and Parkinson’s Disease^6, 17, 18^. Since the lifetime of ROS in the tissue is very short^19^, the effect of H_2_ on quenching of ROS is through to be limited. If H_2_ could be made to more effectively reduce the formation of disulfide bonds between SH groups, H_2_ could more efficiently reduce ROS-induced damage. Among human tissues, the skin is very prone to damage, thus the present study focused on the effect of treating human skin cells, keratinocytes (KC), with H_2_ to protect against ROS-induced damage. Specifically, the protective effect of H_2_ treatment against ROS-induced dysfunctional disulfide bond formation and recovery Ca^2+^ signaling was examined. The data demonstrated that aging processes in KC was found to be selectively oxidized IP_3_Rs, especially IP_3_R1-mediated Ca^2+^ signaling by inducing the formation of H_2_O_2_-mediated disulfide bonds in the skin. In addition, a major protective effect of H_2_ was to reduce disulfide bond formation in the protein caused by oxidative stress, and not by eliminating the generation of ROS in the skin.

## Results

### Reduced Ca^2+^ signaling in KC by H_2_O_2_

**Figure 1:**
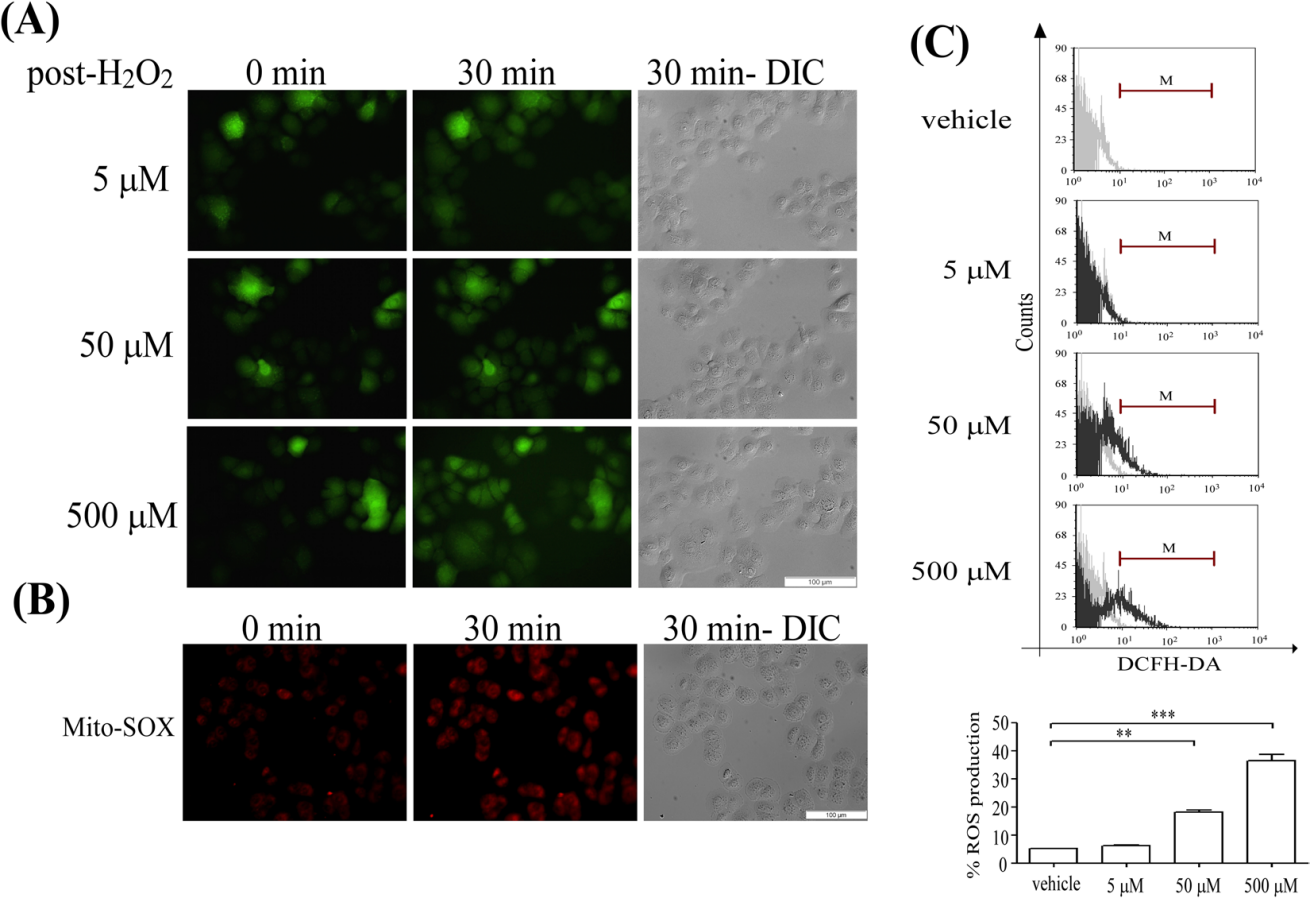
H_2_O_2_ dose-dependently induced oxidative stress in KC. ROS generation was stimulated by 5 μM, 50 μM, and 500 μM H_2_O_2_ for 30 min at 37 °C in humidified 5% CO_2_ and visualized using DCFH-DA or MitoSOX pre-staining. ROS are indicated by the fluorescent signals of (**A**) DCFH-DA or (**B**) MitoSOX, and cell morphology is shown using differential interference contrast (DIC) microscopy. Scale bars in A and B = 100 µm. (**C**) Quantification of ROS production from (**A**) was analyzed using flow cytometry after incubation with H_2_O_2_. Each group (black line) was compared with vehicle (gray line) and ROS production was quantified (lower panel) by adding all black area and subtracting all gray area within the period marked by the red line “M”, using FCS Express 4 Image Cytometry Software (De Novo Software) (**P < 0.01; ***P < 0.005).

**Figure 2:**
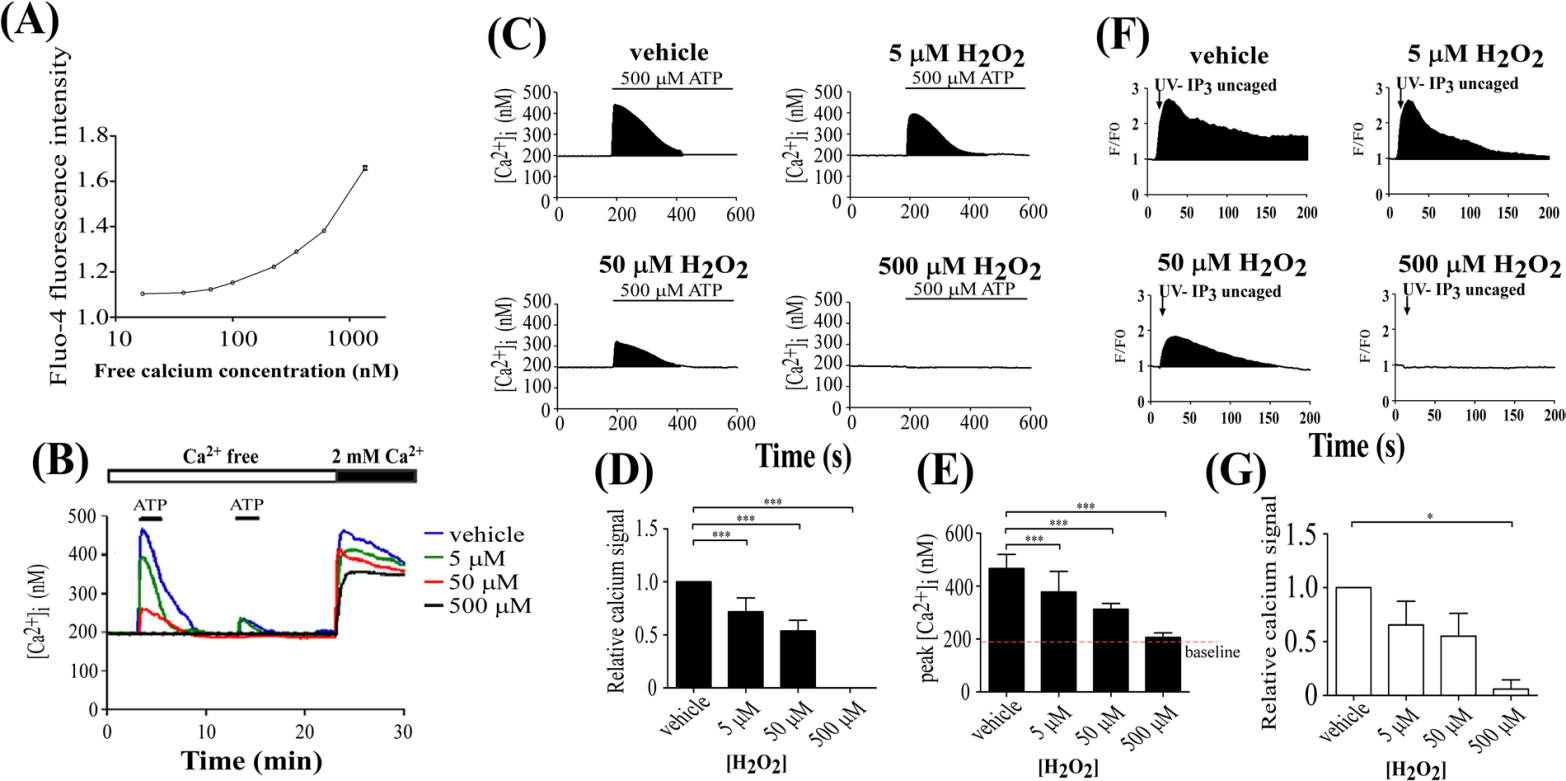
H_2_O_2_ dose-dependently inhibited Ca^2^+ signaling in KC induced by ATP or IP3 uncaging. (**A**) Ca^2+^ calibration curve. Horizontal axis indicates the free concentration of Ca^2+^ ([Ca^2+^]_i_) in standard buffer solution. Vertical axis indicates the ratio of Fluo-4 fluorescence intensity relative to that of baseline fluorescence. (**B**) Effect of H_2_O_2_ on ATP-induced Ca^2+^ signal. Ca^2+^ imaging analysis of the ATP-induced Ca^2+^ response after 30 min pretreatment with vehicle (blue), 5 μM (green), 50 μM (red), and 500 μM (black) H_2_O_2_. Ca^2+^ signals represent the mean value of 20 cells. Twenty-three minutes after two applications of ATP (small black bars) at 10 min intervals in Ca^2+^-free BSS solution (open bar), CaCl_2_ was applied extracellularly (large black bar) to increase Ca^2+^ from 0 to 2 mM to open the store-operated Ca^2+^ channels (N = 5). (**C**) The Ca^2+^ signal from the first simulation with ATP in (**B**) is shown. (**D**) Relative Ca^2+^ signals were measured by calculating the black areas under the curves of the intracellular Ca^2+^ responses with or without H_2_O_2_ treatment in (**C**) (***P < 0.005 in D and E). (**E**) Quantification of the peak ATP-induced Ca^2+^ elevation for all groups in (**C**). (**F**) Effect of H_2_O_2_ on the IP_3_-induced Ca^2+^ response. The concentration of H_2_O_2_ used is indicated. F/F0 expresses the Fluo-4 fluorescence (**F**) relative to baseline fluorescence (F0), which corresponds to changes in the intracellular Ca^2+^ concentration. Uncaging of caged-IP_3_ using UV irradiation released IP_3_ molecules in the cell. The black area under the curve indicates the amount of Ca^2+^ released by uncaged IP_3_ (N = 5). (**G**) The amount of Ca^2+^ was calculated by quantifying the black areas under the Ca^2+^ curve (*P < 0.05).

To examine the types of signaling molecules affected by aging in skin cells, different concentrations of H_2_O_2_ were used to mimic the aging process induced by ROS accumulation. Approximately 10^5^ KC were plated onto 24-mm coverslips in 3.5-cm^2^ dishes, incubated for 2 days, pre-stained with 20 μM 2′,7′-dichlorofluorescein-diacetate (DCFH-DA), then treated with 5 μM, 50 μM, or 500 μM H_2_O_2_. Imaging of the DCFH-DA staining showed ROS generation in the cells, as evidenced by an increase in fluorescence intensity, and dose-dependent effects of H_2_O_2_ exposure were observed with time-lapse recordings (Fig. 1A). The cell morphology was not significantly different between groups, based on differential interference contrast imaging (Fig. 1A). Similar results were observed with 5 μM MitoSOX (Molecular Probes) staining (Fig. 1B) and quantification by flow cytometry (Fig. 1C), which showed that approximately 40% of the cells produced ROS following 500 μM H_2_O_2_ exposure, 20% after 50 μM H_2_O_2_ exposure, and 5% following 5 μM H_2_O_2_ exposure (Fig. 1C). A Ca^2+^-concentration calibration curve was determined (Fig. 2A). H_2_O_2_ decreased ATP (stimulator)-induced Ca^2+^ elevation and store-operated Ca^2+^ (SOC) influx in a dose-dependent manner in KC (Fig. 2B). H_2_O_2_ likely affected ATP-induced Ca^2+^ release via the P2Y receptor and then altered SOC channel-mediated Ca^2+^ entry. To identify the intracellular Ca^2+^ dynamic changes in detail, the ATP-induced Ca^2+^ signal was estimated by measuring the black area under the curve (Fig. 2C). Relative to the response to vehicle, the Ca^2+^ signal was reduced to 72 ± 13% in the 5 µM H_2_O_2_ group and to 54 ± 10% in the 50 µM H_2_O_2_ group, while the peak in each group was estimated to be 433 ± 43 nM for vehicle, and 383 ± 75 nM for 5 µM H_2_O_2_ treatment and 245 ± 21 nM for 50 µM H_2_O_2_ treatment (Fig. 2D,E). Interestingly, ATP stimulation did not induce Ca^2+^ elevation in KC treated with 500 μM H_2_O_2_ (Fig. 2C). Here the baseline corresponded to 185 ± 23 nM of Ca^2+^.

As previously reported, increased phosphorylation of IP_3_Rs suppresses ATP-induced Ca^2+^ release and SOC channel-mediated Ca^2+^ entry^20^. Therefore, we examined whether H_2_O_2_ enhanced IP_3_Rs phosphorylation levels using caged IP_3_. Application of uncaged IP_3_ resulted in Ca^2+^ elevation in Ca^2+^-free BSS buffer without involvement of PKC activation; this Ca^2+^ elevation was reduced by exposure to H_2_O_2_, indicating that H_2_O_2_ interrupted IP_3_Rs function (Fig. 2F). The total Ca^2+^ response is represented by a bar chart, quantified by measuring the black areas under the Ca^2+^ response curves (Fig. 2G). Compared with vehicle, H_2_O_2_ dose-dependently reduced the uncaged IP_3_-induced Ca^2+^ signaling, and the Ca^2+^ response was almost completely blocked by 500 µM H_2_O_2_ (Fig. 2G). The rate of Ca^2+^ decay was similar between the ATP- and uncaged IP_3_-induced Ca^2+^ responses (Fig. 2C,F). These findings indicate that H_2_O_2_ reduced IP_3_Rs functionality, resulting in the suppression of Ca^2+^ signaling due to oxidative stress. Therefore, the possibility of increased phosphorylation of IP3Rs can be excluded as a mechanism underlying the H_2_O_2_-dependent reduction in the Ca^2+^ response in KC.

### ATP-induced Ca^2+^ signaling was inhibited by disulfide bond formation in KC IP_3_Rs

**Figure 3:**
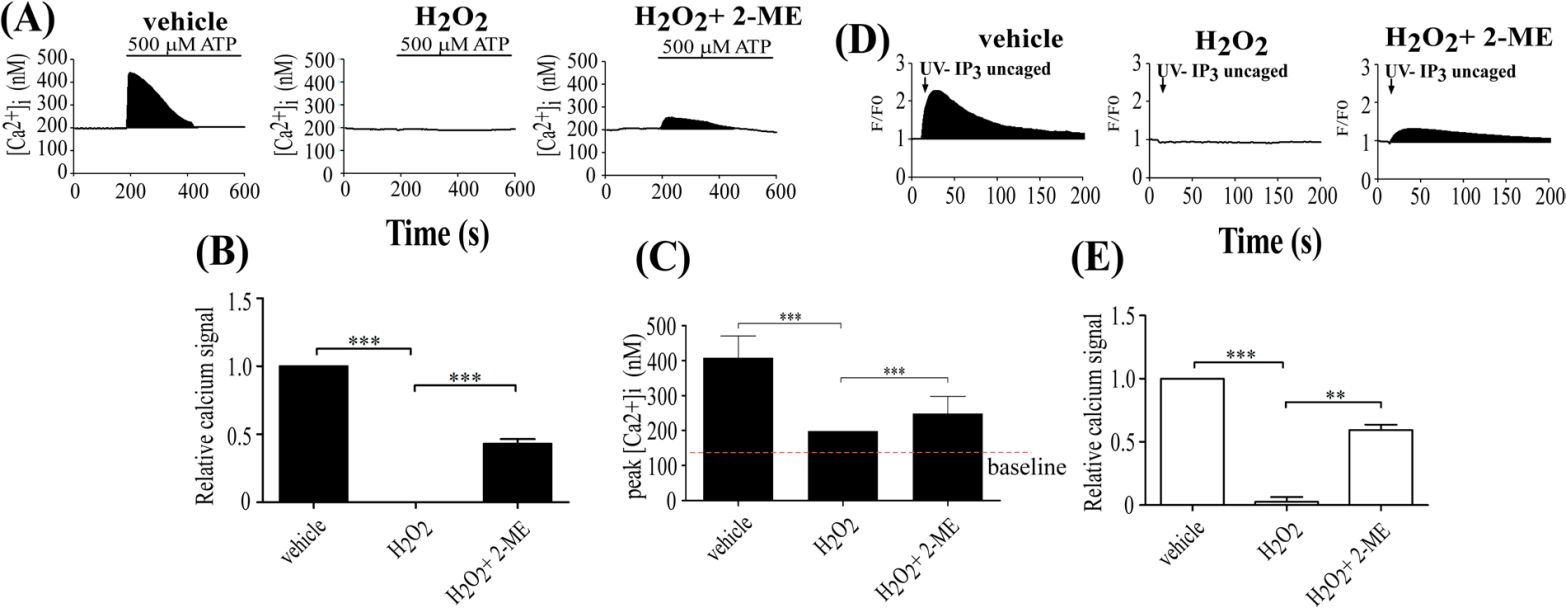
β-mercaptoethanol (2-ME) restored Ca^2^+ response in KC in exposure to H_2_O_2_. (**A**) Ca^2+^ signal in each group (pretreatment with vehicle, 500 μM H_2_O_2_, and 500 μM H_2_O_2_ + 2-ME) was induced by addition of ATP (black bars). (**B**,**C**) Quantification of the black areas representing ATP-induced Ca^2+^ signals (**B**) and the peak of the intracellular Ca^2+^ responses (**C**) shown in (**A**) (N = 3). (D) Ca^2+^ signal in each group (pretreatment with vehicle, 500 μM H_2_O_2_, 500 μM H_2_O_2_ + 2-ME) was stimulated by photolysis-induced uncaging of caged-IP_3_. (**E**) Quantification of the black areas in (**D**) represent the amount of Ca^2+^ released by the uncaged IP_3_ (*P < 0.05; **P < 0.01; ***P < 0.005).

Because the suppression of the uncaged IP_3_-induced Ca^2+^ elevation did not result from IP_3_Rs phosphorylation, we hypothesized a novel model to explain the findings. It is worth noting that oxidative stress can induce disulfide bond formation, thus impairing molecular chaperoning, translation, glycolysis, cytoskeletal structure, cell growth, and signal transduction^10^. That H_2_O_2_ can oxidize cysteine or methionine sulfhydryl groups to sulfenic acid or disulfide bonds^8, 9^ raised the possibility that H_2_O_2_ decreased the Ca^2+^ signal by eliciting IP_3_Rs disulfide bond formation. This possibility was examined using the reduction agent β-mercaptoethanol (2-ME; Sigma-Aldrich). ATP-induced Ca^2+^ elevation was completely inhibited by 500 μM H_2_O_2_ (Fig. 3A,B), but the Ca^2+^ response was partially recovered, increasing from 0% to 43 ± 3.5% when 10 mM 2-ME and H_2_O_2_ were applied together (500 μM H_2_O_2_ + 2-ME; Fig. 3A,B). 2-ME increased the Ca^2+^ peak from 198 nM (500 μM H_2_O_2_) to 248 (500 μM H_2_O_2_ + 2-ME; Fig. 3C). To further examine the effect of H_2_O_2_ on IP_3_Rs, caged IP_3_ was utilized to detect whether only IP_3_Rs functionality was reduced by H_2_O_2_-induced disulfide bond formation. As expected, the Ca^2+^ released in response to uncaged IP_3_ stimulation was largely recovered, increasing from 3 ± 3% to 62 ± 5% (500 μM H_2_O_2_ + 2-ME) relative to vehicle, when 2-ME was applied with H_2_O_2_ (Fig. 3D,E). When 2-ME and H_2_O_2_ were applied together, however, the H_2_O_2_-induced decrease was protected to some extent (Fig. 3D). Thus, it is possible to determine 500 μM H_2_O_2_-induced reduction of the Ca^2+^ signal was dependent on disulfide bond formation in IP_3_Rs. These results suggest that disulfide bond formation in IP_3_Rs induces a conformational change and obstructs IP_3_ binding to IP_3_Rs under oxidative stress. Because the aging process is associated with both oxidative stress and Ca^2+^ deficiency^14^, it is reasonable to suggest that ROS-induced protein disulfide bond formation may contribute to skin aging.

### H_2_ gas-containing media protected KC against H_2_O_2_-induced disulfide bond formation in IP_3_Rs

Recently, H_2_-containing medium was reported to protect against ROS-induced damage by buffering the effects of oxidative stress or superoxide formation^21^. H_2_ was further investigated to determine whether it protects against H_2_O_2_-oxidized IP_3_Rs function and reduction of the resulting Ca^2+^ signal. Preparation and how to use H_2_-containing BSS is described in the Materials and Methods section. The H_2_-containing BSS was stored in an open glass bottle, and the hydrogen concentration reduction in the media was measured every hour for 12 h using a hydrogen-sensitive electrode. The half-life of H_2_ was estimated to be approximately 6 h in BSS (Fig. 4A). Because the lower limitation of effective H_2_ concentration was estimated as 0.08 ppm^18^, all the experiments were designed to finish within a few hours. To examine the effect of H_2_-BSS on H_2_O_2_-decreased IP_3_Rs function, we evaluated the following four groups: vehicle, 500 μM H_2_O_2_, 500 μM H_2_O_2_ with 2-ME, and 500 μM H_2_O_2_ with H_2_ in BSS buffer. Ca^2+^ release in KC was induced with the addition of 500 μM ATP. Both the 500 μM H_2_O_2_ with 2-ME and the 500 μM H_2_O_2_ with H_2_ groups produced similar results, in that the Ca^2+^ was 65 ± 14% (500 μM H_2_O_2_ + 2-ME) or 79 ± 12% (500 μM H_2_O_2_ + H_2_) relative to that of the vehicle group (Fig. 4B,C) and peaked at 385 ± 45 nM (500 μM H_2_O_2_ + 2-ME) or 407 ± 73 nM (500 μM H_2_O_2_ + H_2_) (Fig. 4B,D). There was negligible Ca^2+^ response with exposure to 500 μM H_2_O_2_ alone (Fig. 4B,C). To further evaluate the effect of H_2_-BSS on 500 μM H_2_O_2_-induced IP_3_Rs dysfunction, uncaged IP_3_ was tested to confirm whether ROS selectively affected IP_3_Rs. As expected, IP_3_Rs function was partially protected by treatment with H_2_. In the focal IP_3_ uncaging experiment, the IP_3_-mediated Ca^2+^ signal in 2-ME treated cells was 71 ± 7% (500 μM H_2_O_2_ + 2-ME) that of vehicle and the Ca^2+^ signal of H_2_-treated cells was 80 ± 3% (500 μM H_2_O_2_ + H_2_; Fig. 4E,F). These results suggest that H_2_-containing BSS protected the ATP-induced Ca^2+^ signal in skin by reducing the H_2_O_2_-induced disulfide bonds in IP_3_Rs and had more effective than 2-ME in restoring H_2_O_2_-induced Ca^2+^ suppression.

**Figire 4:**
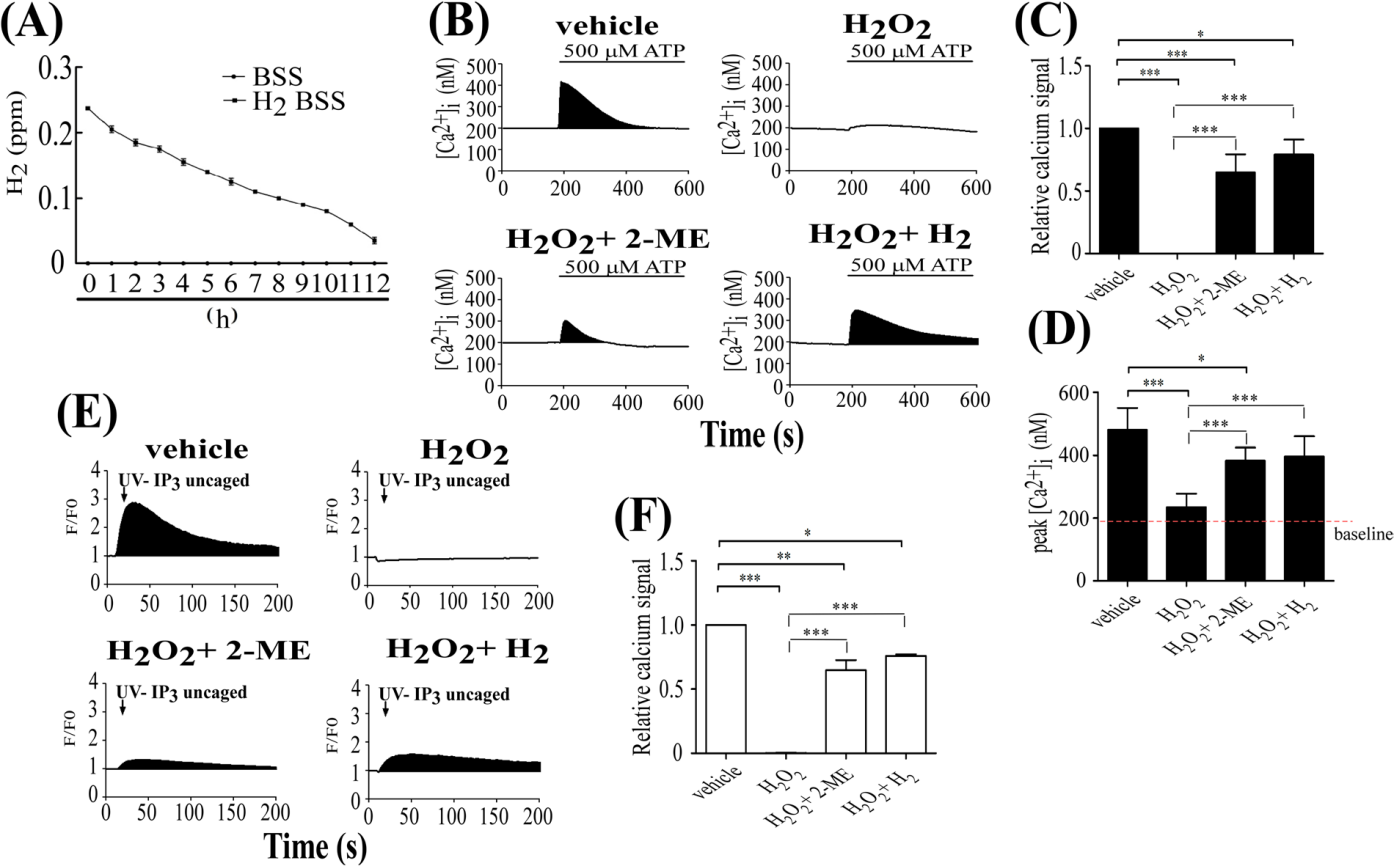
H_2_ was more effective than 2-ME in restoring H_2_O_2_-induced Ca^2^+ suppression in KC. (**A**) Time-dependent reduction of H_2_ content in BSS buffer. (**B**) Mean Ca^2+^ response to ATP application in each group: vehicle, 500 μM H_2_O_2_, 500 μM H_2_O_2_ + 2-ME, and 500 μM H_2_O_2_ + H_2_. (**C**) The Ca^2+^ response was quantified by measuring the black areas under the Ca^2+^ curve. (**D**) The peak of ATP-induced intracellular Ca^2+^ signal (N = 5). (**E**) Protective effect of H_2_-BSS on H_2_O_2_-induced reduction of the uncaged IP_3_-stimulated Ca^2+^ response. Ca^2+^ signals are shown as black areas after uncaged IP_3_ stimulation. (**F**) Quantification of Ca^2+^ signals by measuring the black areas under the Ca^2+^ response curve (N = 5) (*P < 0.05; **P < 0.01; ***P < 0.005).

### H_2_-BSS protected against H_2_O_2_-induced damage of IP_3_R1 by reducing disulfide bond formation, not by quenching ROS in KC

**Figure 5:**
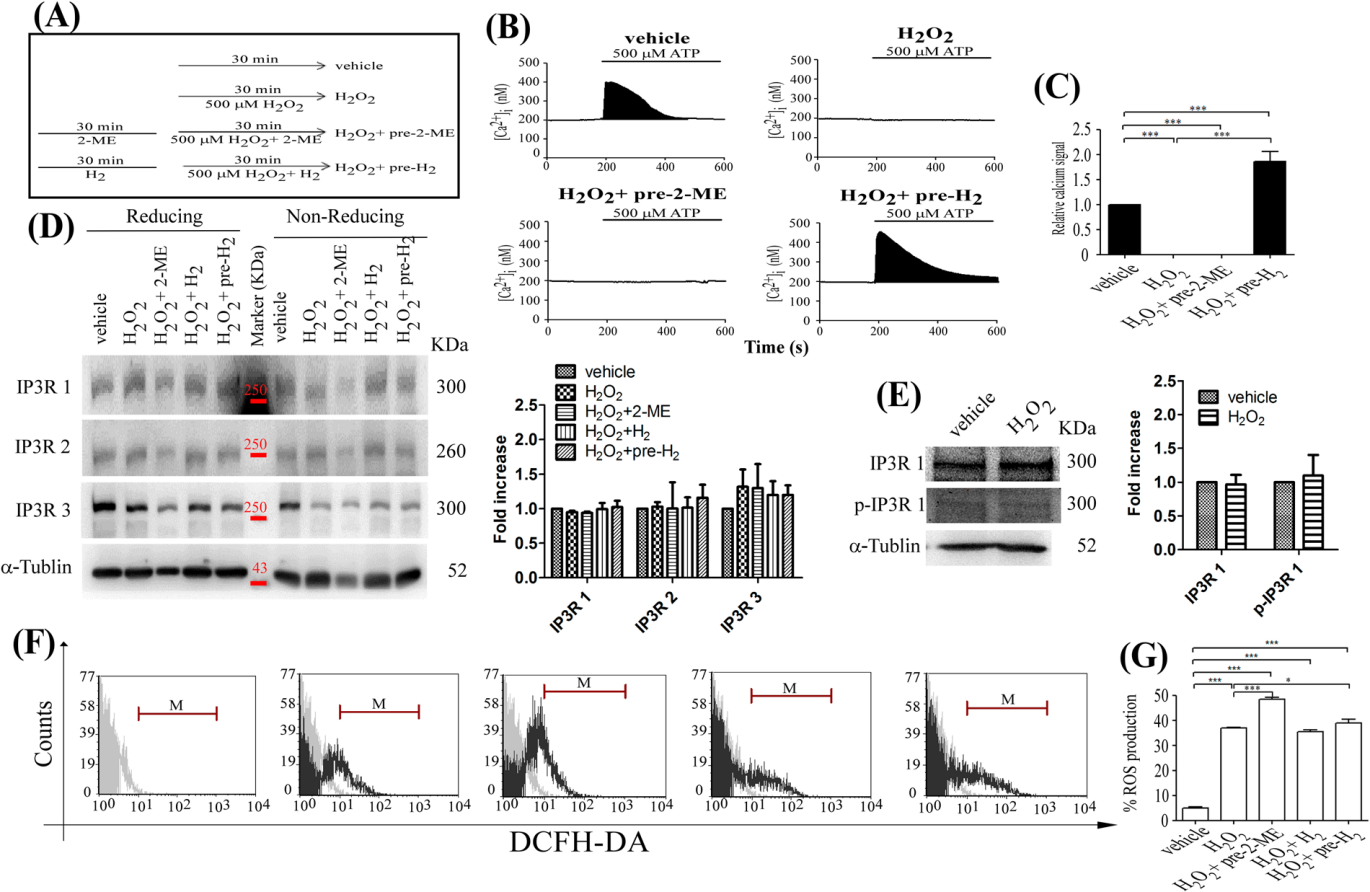
H_2_ decreased disulfide bond formation but not ROS production in H_2_O_2_-exposed KC. (**A**) Effect of pretreatment with 2-ME-BSS or H_2_-BSS on the H_2_O_2_-induced reduction of ATP-induced Ca^2+^ signal in KC. Experimental design for treatment in each group: vehicle, 500 μM H_2_O_2_, 500 μM H_2_O_2_ + pre-2-ME, and 500 μM H_2_O_2_ + pre-H_2_. Ca^2+^ signal was induced by ATP stimulation in each group (**B**) and calculated by the black-colored areas under the Ca^2+^ curve (N = 3) (***P < 0.005) (**C**). (**D**) Expression of IP_3_Rs under non-reduced and reduced conditions is shown using SDS-PAGE and Western blot. Protein extracts from each group were resolved in non-reducing (without 2-ME) SDS-PAGE (7% gel), transferred onto Hybond-P polyvinylidene fluoride membranes, and incubated with antibodies against IP_3_R1, IP_3_R2 and IP_3_R3 and α-Tublin. As a control, the lysates were reduced using 100 mM 2-ME (reduced; right lanes). Proteins that form disulfide bonds exhibit faster migration and thus appear as lower molecular weight bands. Quantify of IP_3_Rs expression in reduced gel as shown in right panel in represent the average of three independent experiments. (**E**) Western blot analysis of the expression of IP_3_R1 and phosphorylated IP_3_R1 (p- IP_3_R1) after incubation of the cells with or without 500 μM H_2_O_2_. Treatment with 500 μM H_2_O_2_ had no effect on the density of the bands for phosphorylated-IP_3_R1 (p-IP_3_R1), IP_3_R1 or α-Tublin. Quantify of expression in IP3R1 and p-IP3R1 as shown in right panel in represent the average of three independent experiments. (**F**) Flow cytometry was used to estimate the (**G**) ratio of ROS generation in each group (*P < 0.05; ***P < 0.005).

Since 2-ME-BSS and H_2_-BSS had different effects on 500 μM H_2_O_2_-induced damage of Ca^2+^ release with IP_3_Rs in KC, we performed additional experiments to make the difference between the effects of H_2_ and 2-ME. Briefly, cells were pretreated with 2-ME or H_2_ gas for 30 min and compared (Fig. 5A). Protection of the Ca^2+^ signaling system following pretreatment with H_2_-BSS (500 μM H_2_O_2_ + pre-H_2_) was 185 ± 19% that following pretreatment with vehicle (Fig. 5B and C), while no Ca^2+^ signal was observed following pretreatment with 2-ME (500 μM H_2_O_2_ + pre-2-ME) (Fig. 5B). The enormous difference between the effects of pre-treatment with H_2_-BSS and 2-ME may be due to the different mechanisms by which they reduce disulfide bond formation in IP_3_Rs. 2-ME is a strong reducing agent that reduces all proteins in the cell, resulting in considerable conformational changes and thereby dysfunction of all functional proteins. In order to confirm this possibility, we examined if pretreatment with 2-ME could reduce all proteins to the extent that no Ca^2+^ signal can be induced by ATP stimulation. Additional assays were performed to confirm whether H_2_-BSS protected against H_2_O_2_ damage by reducing disulfide bond formation. In this experiment, non-reducing sodium dodecyl sulfate-polyacrylamide gel electrophoresis (SDS-PAGE) and Western blot analysis were performed as described previously^10^. We focused on examining the formation of disulfide bonds of IP_3_Rs with three subtypes: IP_3_R1, IP_3_R2 and IP_3_R3 via treating the specific antibodies^20, 22, 23^. All IP3Rs display the molecular weight over 250 kDa. As shown in Fig. 5D, IP_3_R1 with disulfide bonds, as detected by IP_3_R1 antibody and migration in a broad band secondary to oxidized S-S formation. However, IP_3_R2 and IP_3_R3 did not present clear disulfide bonds formation based on the similar molecular weight of broad band (Fig. 5D). The formation of disulfide bonds affects the conformation and electrophoretic mobility of redox-sensitive proteins^24–26^. Proteins forming intra-molecular disulfide bonds exhibit distinct types of migration in non-reducing (without 2-ME in protein loading buffer) SDS-PAGE and reducing SDS-PAGE (with 2-ME in protein loading buffer)^10^. As expected, KC exposed to H_2_O_2_ had IP_3_R1 containing disulfide bonds, as evidenced by the lower broad band when electrophoresed under non-reducing conditions, but KC treated with a reducing agent (2-ME, H_2_ or pre- H_2_) had a higher band and lightly stained bands similar to the vehicle-treated KC (Fig. 5D). Western blot analysis also confirmed no effect on the level of IP3R1 phosphorylation (Fig. 5E). This finding suggests that the reducing agents potentially altered the conformation of the IP_3_R1 by breaking disulfide bonds, yet neither reducing agent completely reduced IP_3_R2 and IP_3_R3 disulfide bonds in the cell. This result explains why the IP_3_Rs-mediated Ca^2+^ signal was not completely protected with application of 2-ME or H_2_. These reducing agent may not be able to reach inside of protein structure, this was conjoined by prolonged treatment by H_2_-BSS for 30 more minutes, as shown in Fig. 5B and C. As previously established, superoxide dismutase converts superoxide anion radicals into H_2_O_2_, which is detoxified into H_2_O by either glutathione peroxidase or catalase^27^. Our examination of ROS production using flow cytometry revealed that neither H_2_-BSS nor 2-ME reduced ROS production induced by 500 μM H_2_O_2_ in KC (Fig. 5F and G). The finding that H_2_O_2_ was not detoxified with H_2_ is not similar to the results of a previous study, which showed that H_2_ reduces hydroxyl radicals ^**∙**^HO^17^, because H_2_O_2_ can be metabolized into$\cdot {\rm{HO}}$ via catalysis^28^.

**Figure 6:**
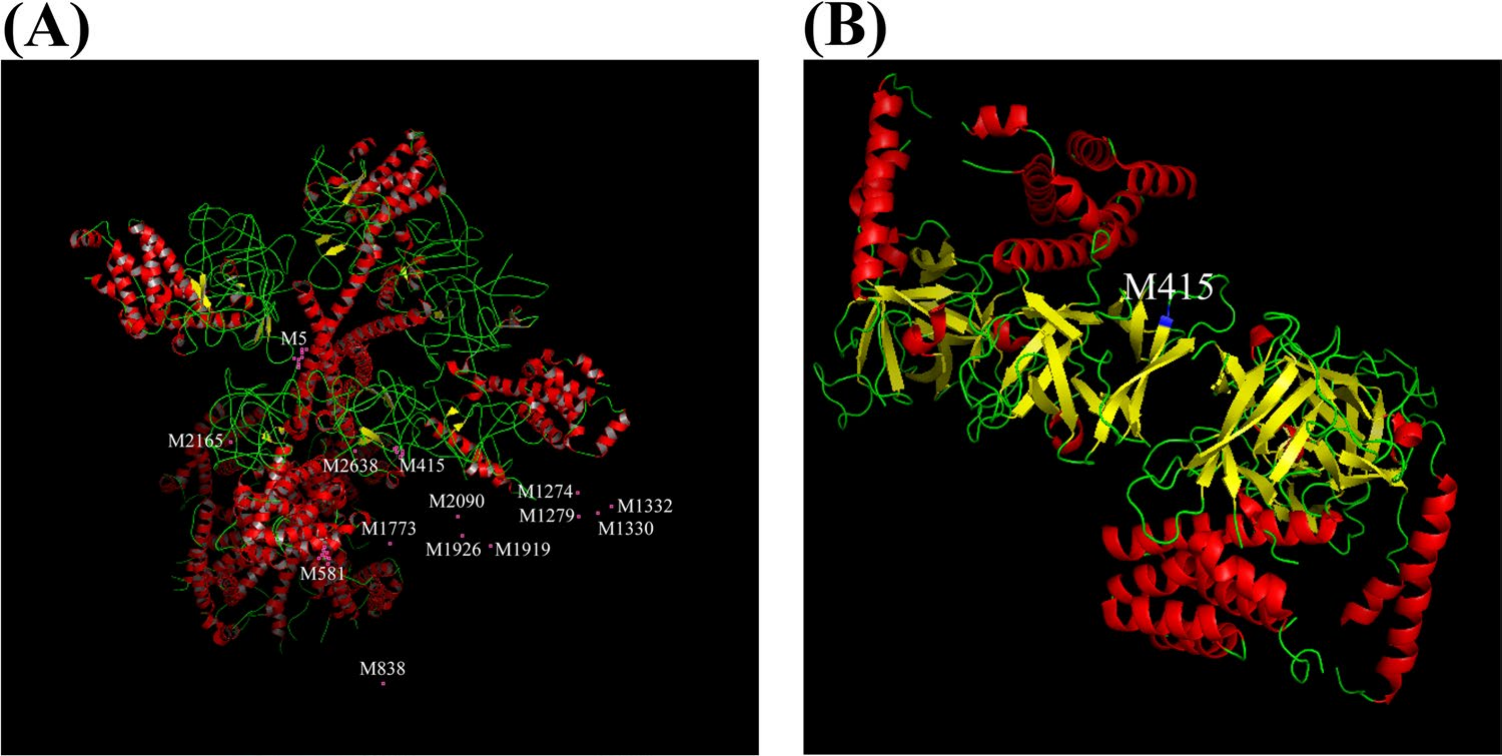
The location of each MBP-modified methionine in IP3R1 structure. (**A**) Cryo-EM imaging of IP3R1 displays the sheets (yellow), helixes (red) and loops (green); each MBP-modified methionine listed Table 1 is labeled with pink spot in IP3R1. However, majority of MBP-modified Mets are constructed by hydrogen linkages (pink spots in dark empty spaces). Met-5, Met-415 and Met-581 are located in loop and sheet respectively. (**B**) Crystal structure of IP3 binding domain from IP3R1 shown in Met-415 (blue) is in the boundary between sheet and loop. Both Cryo-EM imaging and crystal imaging have been used to represent the information from Protein Data Bank (PDB) and the 3D structure built using PyMOL software.

To detect the formation of disulfide bonds in IP3R-1, we employed liquid chromatography tandem-mass spectrometry (LC-MS/MS). The sulfhydryl residue Cys or Met, through which disulfide bridges can be produced by H_2_O_2_, is labeled with maleimide-PEO_2_-biotin (MPB)^29^. We examined whether MPB was applicable to assay H_2_O_2_-induced intermolecular disulfide bond formation in receptor protein-tyrosine phosphatase α (RPTPα). MPB-containing peptides signal located the intermolecular disulfide bond to the Cys-723 of RPTPα, and the counts of H_2_O_2_-induced MPB-containing peptides was higher than the counts in the group of vehicle or H_2_O_2_ with 2-ME (Supplementary Table S1). However, identification of the disulfide bonds formation in Cys residues with delta mass 646.24 (Cys+MPB) can’t search the MPB-modified Cys peptides. It could be either the tiny signal of MPB-modified Cys difficult detection by LC-MS/MS, or Cys is not the major residue in H_2_O_2_-induced disulfide bonds formation. Consequently, we demonstrated whether H_2_O_2_ elicited disulfide bridges formation in methionine-containing peptides. As shown in Table 1, MPB-modified residue locates in methionine and has mostly increased level of counts in H_2_O_2_ treatment. H_2_ partially recovered (decreased) the counts of MPB-containing peptides (Table 1). Besides, we attempted to recognize each MPB-modified methionine in IP3R1 protein via Cryo-EM and crystal structure models, which were established from Fan *et al*. and Seo *et al*. respectively^30, 31^. In this huge IP3R1 3D structure, most of MPB-modified methionines located in hydrogen-bonding networks not in functional domains (Fig. 6A); Met-5, Met-415 and Met-581 located in loop and sheet, but only Met-415 located in IP3 binding domain, the boundary between sheet and loop (Fig. 6B). H_2_ treatment restrained the level of counts in MPB-modified Mets, explained why H_2_ performed the Ca^2+^ signal significant recovery due to reduction of disulfide bonds formation.

**Table 1:** Presentation of proteomics analysis from the methionine-labeling peptides in IP3R1.

IP3R1	Average normalized abundances (counts)				
Sequence	vehicle	H_2_O_2_	H_2_O_2_ + 2-ME	H_2_O_2_ + H_2_	H_2_O_2_ + pre-H_2_
AYMQGEVEFEDGENGEDGAASPR (Met-2090)	2383.54	3257.5	3069.95	3436.03	3190.64
EEEKPVMLK (Met-415)	6420.76	7949.82	5462.67	4058.18	6508.37
EGASNLVIDLIMNASSDR (Met-1773)	2459.76	3336.82	2769.74	2373.98	2846.81
DDLEMSAVITIMQPILR (Met-1919, Met-1926)	10500	10500	8721	10300	12000
KCQDMVMAELVNSGEDVLVFYNDR (Met-1330, Met-1332)	17500	15600	16900	15300	18800
HINLFLNPGILEAVTMQHIFMNNFQLCSEINER (Met-1274, Met-1279)	3620.65	4325.82	3111.94	2176.18	3044.65
FAQTMEFVEEYLRDVVCQR (Met-838)	6724.08	8410.16	6306.69	8136.91	7099.21
MSSFLHIGDICSLYAEGSTNGFISTLGLVDDR (Met-5)	4937.13	3942.33	4739.32	4453.46	4029.86
NLDWFPRMR (Met-2638)	1726.98	1696.6	1752.37	1450	1467.72
NQEYIAKQFGFMQK (Met-581)	18200	24300	20200	26800	29100
TMEQIVFPVPSICEFLTKESK (Met-2165)	119000	91500	110000	111000	101000

LC-MS/MS analyzed MBP-modified peptide sequences with delta mass 674.45 (Met + MBP) and isotopic quantification. Average normalized abundance reveals signal counts from each peptide with three tests. The modified Met residues are indicated by residue number in IP3R1.

## Discussion

The findings of the present study reveal the potential association of the skin aging process with Ca^2+^ signal dysfunction via the formation of H_2_O_2_-induced disulfide bonds^10^ in IP_3_Rs that lead to selective damage of the IP_3_Rs. Co-treatment with 2-ME and 500 μM H_2_O_2_ decreased the disulfide bond formation, evidenced by the protection of ATP-induced Ca^2+^ release in KC with 2-ME. This provides clear evidence that oxidative stress-related skin damage is associated with Ca^2+^ signaling defects, similar to 2-ME, H_2_ gas in BSS decreased IP_3_R1 disulfide bond formation due to ROS exposure but not IP_3_R2 and IP_3_R3. The major function of H_2_ in BSS in skin was the protection of ATP-induced Ca^2+^ signaling, but not by direct quenching of ROS. ATP is thought to be an external 1^st^ cell-to-cell communication around the other cells equipped with GPCR. Because BSS media has no Cu and Fe to make hydroxyl radical, contribution of hydroxyl radical will be smaller than usual when the cells are treated by H_2_O_2_. Previous reports indicate that the presence of reducing agents decreased number of disulfide bonds, resulting in a loss of cross-link-induced stability produced by the chemical microenvironment^32^. It is reasonable to assume that the skin aging process is associated with ROS accumulation in the skin, which leads to IP_3_Rs dysfunction by inducing conformational changes due to disulfide bond formation.

Recent study has pointed out the treatment of H_2_ in acute erythematous skin diseases, which are associated with large ROS accumulation in KC^33^. H_2_ improved significantly in erythema of these patients and did not affect physiological parameters and deterioration of the blood chemistry^33^. Although elimination of ROS produced by H_2_O_2_ is also performed in H_2_, our study firstly indicates H_2_ also reduces disulfide bond formation and restores protein function. However, our finding revealed IP_3_R1 but not IP_3_R2 and IP_3_R3 is recovered the function by H_2_ treatment. It may be possible due to the different distribution of S-S bond formation in each subtype of IP_3_Rs. H_2_O_2_ and H_2_ independently oxidize and/or reduce each subtype of IP_3_Rs; therefore, the exposed site (Cys or Met) of each subtype of IP_3_Rs could be affected by H_2_O_2_ and H_2_ independently or correlatively. This is reasonable to explain why IP_3_R1 displays more obvious in disulfide bond formation and is more efficient for H_2_ reduction.

Besides, H_2_ acts also as a reducing agent at a lower concentration, but the amount of H_2_ was sufficient to disrupt metabolic oxidation reducing reactions or to interrupt ROS-induced disruption of cell signaling^17^. H_2_ induces superoxide dismutases (SODs) and heat shock proteins (HSPs) activity to quench ROS production^6^ and may thereby completely protect the IP_3_Rs Ca^2+^ signal. Nevertheless, pretreatment with H_2_ failed to decrease H_2_O_2_-induced ROS production. Another possible mechanism underlying the H_2_-induced elimination of ROS damage of IP_3_Rs is the activation of glutathione/thioredoxin systems, which reduces H_2_O_2_-induced disulfide bond formation. To date, however, it is not clear whether H_2_ facilitates glutathione/thioredoxin system activity, although studies have reported that glutathione/thioredoxin systems are involved in the modulation of disulfide bond formation during oxidative stress^10, 34^.

In the skin aging process, UV irradiation increases ROS production^6, 35^. Superoxide radicals (^**∙**^O_2_^−^), hydroxyl radicals (^**∙**^HO), and peroxyl radicals (LOO^**∙**^), singlet oxygen (^1^O_2_), and hydrogen peroxide (H_2_O_2_) are involved in UV-stimulated oxidative stress^6^. According to a previous report^17^, however, H_2_ selectively reduces the hydroxyl radical, yet it is not involved in the efficient elimination of oxidative-stress-induced damage due to UV irradiation. The results of the present study indicate that, rather, a major mechanism underlying H_2_ effects is to protect IP_3_Rs-mediated Ca^2+^ signaling by reducing ROS-induced disulfide bond formation. IP_3_Rs-mediated Ca^2+^ signaling has a central role in several physiologic processes, such as fertilization, proliferation, muscle contraction, cell metabolism, vesicle and fluid secretion, and information processing^11^. As aging-induced dysfunction of Ca^2+^ signaling leads to cell death or genome instability, H_2_ can protect skin cells from destruction by maintaining the Ca^2+^ signal and thus preserving normal physiologic conditions. The results of the present study clearly demonstrate the role of H_2_ in protecting against the aging process in skin and provide evidence to support further investigation of the clinical applicability of H_2_.

## Methods

### Cell culture

Human primary KC were isolated from foreskins obtained via routine circumcision as described previously^36^. The skin specimens were washed with phosphate-buffered saline (PBS), cut into small pieces, and cultured in medium containing 0.25% trypsin (Gibco BRL) overnight at 4 °C. The epidermal sheet was lifted from the dermis utilizing fine forceps. The epidermal cells were then pelleted using centrifugation (500 g, 10 min) and separated into individual cells by repeated aspiration. The KC were incubated in serum-free KC growth medium, supplemented with human recombinant epidermal growth factor and bovine pituitary extract (5 μg/mL each), insulin (5 μM/mL), and hydrocortisone (5 μg/mL; Gibco BRL) at 37 °C in humidified 5% CO_2_, and supplemented with KC growth medium for five generations^20, 37^.

### Calcium imaging

The intracellular Ca^2+^ response was induced by application of ATP (Sigma-Aldrich), according to methods previously described^20^. Before the experiments, cells were stained with 1 μM Fluo-4-AM (Molecular Probes) at 37 °C for 20 min and then washed with BSS buffer (5.4 mM KCl, 5.5 mM d-glucose, 1 mM MgSO_4_, 130 mM NaCl, 20 mM Hepes pH 7.4, and 2 mM CaCl_2_). Intracellular Ca^2+^ concentrations were estimated based on the ratio of fluorescence intensities emitted upon excitation with consecutive 3-s pulses of 488-nm light at a resolution of 1376 × 1038 pixels using an Olympus Cell^R IX81 fluorescence microscope (Olympus) equipped with an MT 20 illumination system (Olympus) and UPLanApo 10 × objective lens. The intracellular Ca^2+^ concentration was estimated based on calibration curves as follows. A Ca^2+^ calibration curve was created using a Ca^2+^ Calibration Buffer kit (Molecular Probes). Intracellular Ca^2+^ ([Ca^2+^]_i_) was calculated from Fluo-4 excited at 488 nm and imaged using an Olympus Cell^R IX81 fluorescence microscope and UPLanApo 10 × objective lens at 20 °C. Fluo-4 signals were calibrated by measuring the fluorescence intensity from microcuvettes containing 10 mM K_2_-EGTA (pH 7.20) buffered to various [Ca^2+^] levels. Ca^2+^ concentration was calculated using the following formula: [Ca^2+^]_i_ = KD *(F − F_min_/F_max_ − F). Plotting the fluorescence intensity versus [Ca^2+^] yielded the calibration curve with the formula of: [Ca^2+^]_i_ = KD *(F − F_min_/F_max_ − F), where KD = 345 nM, F = Fluo-4 intensity, F_max_ = 640, and F_min_ = 21.7 for Fluo-4.

### Focal uncaging

Caged compounds were uncaged to investigate modifications of IP_3_Rs by photolysis using UV light (300–400 nm) as described previously^20^. For photolytic uncaging, an Olympus FV1000 MPE multiphoton laser scanning microscope equipped with an argon laser was used to produce a collimated light beam as the principal uncaging laser line at λ = 408 nm. To detect the effect of H_2_O_2_ (Sigma-Aldrich) on IP_3_Rs in Ca^2+^ pathways, 10^5^ primary KC were plated on 2.4-mm coverslips in a 4-cm dish. For H_2_O_2_ treatment, cells were pretreated with 1 μM caged IP_3_ (Molecular Probes) for 2 h^38^ and stained with the Ca^2+^ dye Fluo-4 to analyze changes in intracellular Ca^2+^ levels. Ca^2+^-induced fluorescence was observed using an Olympus FV1000 laser-scanning microscope. Caged IP_3_ was uncaged by illumination with UV light (λ = 408 nm) and the released IP_3_ molecule was immediately able to bind to the IP_3_Rs.

### Western blot analysis

Western blot analyses were performed utilizing whole-cell lysates. Briefly, cells were lysed by incubating for 30 min on ice in M-PER Mammalian Protein Extraction Reagent (Thermo Fisher Scientific), containing proteinase and phosphatase inhibitors. Cell debris was removed via centrifugation at 10,000 g for 10 min at 4 °C. The protein concentration of cell lysates was determined using the Bradford method (Bio-Rad). Proteins (100 μg) in cell lysates were resolved using sodium dodecyl sulfate-polyacrylamide gel electrophoresis in a 7% gel with or without 2-ME and then transferred to a Hybond-P polyvinylidene fluoride membrane (Amersham Biosciences). The membrane was first incubated with primary antibodies against IP_3_R1 (Cell Signaling Technology), phospho-IP_3_R1 (Cell Signaling Technology), IP_3_R2 (Merck Millipore), IP_3_R3 (BD Biosciences) and α-Tublin (Santa Cruz Biotech), and then with horseradish peroxidase-conjugated secondary antibodies. Immunoreactive proteins were visualized using enhanced chemiluminescence reagents (Amersham Biosciences).

### ROS measurement using flow cytometry

DCFH-MA (Sigma-Aldrich) staining was used to quantify ROS generation from the cells. Briefly, cells were stained with 20 μM DCFH-DA at 37 °C for 20 min and then washed with BSS buffer. The cells were collected after application of H_2_O_2_, reducing agent 2-ME, or H_2_ gas-containing BSS (H_2_-BSS) at 37 °C for 30 min. ROS production was determined using flow cytometry (LSR II, BD) with fluorescence emission at 488 nm.

### Disulfide-bond labeling and in solution digestion

To investigate the intracellular disulfide bonds formation, we modified the labeling protocol from Clive Metcalfe *et al*. study^29^. Briefly, the cells were treated 2.5 mM Methyl-PEO_12_-maleimide (MPM, Thermo Fisher Scientific) with 0.05% TritonX-100 (Sigma-Aldrich) in PBS containing 1% bovine serum albumin (BSA, Sigma-Aldrich) at 4 °C for 30 min; MPM can entry the cell and bind the intracellular free sulfhydryl groups with TritonX-100 treatment. After washing the cells with PBS containing 1% BSA at 25 °C for three times (1 min/time), the cells were reduced with 2.5 mM tris(2-carboxyethyl)phosphine (TCEP, Sigma-Aldrich) and 10 mM dithiothreitol (DTT, Sigma-Aldrich) at 25 °C for 30 min. After washing (1% BSA in PBS) three times, cells were labelled with 2.5 mM Maleimide-PEO_2_-biotin (MPB, Thermo Fisher Scientific) in PBS containing 1% BSA at 4 °C for 30 min, and then cells were washed for three times. Total protein lysates were collected for in solution digestion after adding M-PER protein extraction reagent (Thermo Fisher Scientific).

We utilized nitrogen gas to blow-dry the total protein lysates to protein pellet, and suspended protein pellet with 0.1% RapiGest SF solution (Waters). Protein sample was treated with 20 mM iodoacetamide (IAA, Sigma-Aldrich) in the dark for 30 min and then desalted by using Amicon® Ultra-0.5 Centrifugal Filter Device (Merck Millipore). After incubating with trypsin for peptides digestion overnight, protein sample treated with 1% of formic acid (FA, Sigma-Aldrich) and confirmed pH <2 with a pH paper. The tryptic peptide sample was analyzed disulfide bonds labeling by use of quantitative proteomics techniques utilizing serially coupled lipid chromatography data-independent parallel fragmentation mass spectrometry (LC/MS^E^).

### LC/MS^E^ analysis

Quantitative analysis will be performed essentially on a Waters Xevo G2 qTof mass spectrometer (Waters). In brief, the tryptic peptide sample will be chromatographically separated on M-class UPLC separations module (Waters) incorporating 50 femtomole tryptic digested BSA as the internally spiked protein quantification standard. Peptide elution will be executed through a 75 μm × 25 cm BEH C-18 column (Waters) under gradient conditions at a flow rate of 300 nL/min over 70 min at 40 °C. The mobile phase will be composed of acetonitrile as the organic modifier and formic acid (0.1% v/v) for molecule protonation. Mass spectrometry was performed on Xevo G2 qTof (waters) instrument equipped with a nanoflow electrospray ionization (ESI) interface and operated in the data-independent collection mode (MS^E^). Parallel ion fragmentation will be programmed to switch between low (4 eV) and high (15–45 eV) energies in the collision cell, and data will be collected from 300 to 2000 m/z utilizing glu-fibrinopeptide B (Sigma-Aldrich, m/z 785.8426) as the separate data channel lock mass calibrant. Data will be processed with ProteinLynx GlobalServer v3.0 (waters) for qualification and Progenesis QI for proteomics (Waters) for relative quantification, respectively. Deisotoped results will be searched for protein association and modification from the Uniprot (www.uniprot.org) human protein database.

### H_2_ gas-containing BSS and assessment of H_2_ content

Hydrogen-containing water (H_2_ water) was produced using an Aurora H_2_ water-making machine (Kyoyo Company, Japan). To generate H_2_-containing BSS, First, H_2_ water with one-tenth concentration of BSS was made from sterile water using the Aurora machine. Then, H_2_ water was mixed with BSS stock solution to make final H_2_-containing BSS (pH = 7.4). The H_2_ content was measured using a hydrogen electrode (Kyoyo Company, Japan).

### Ethical approval

Human primary KC from foreskins was approved from the Institutional Review Board/Ethics Committee (IRB) in Kaohsiung Medical University Chung-Ho Memorial Hospital, number KMUH-IRB-960119. All methods were performed in accordance with the relevant guidelines and regulations. Informed consent of all participants was obtained. A total number of 32 samples (foreskins) were collected from 2007 to 2008 of which were further analyzed by isolating KC from skin.

### Statistical analysis

GraphPad Prism (La Jolla, CA) was used to generate bar charts; error bars indicate standard deviations. A one-way, two-tailed analysis of variance (ANOVA) was also utilized to compare means of each group. A P-value of less than 0.05 for differences between groups was considered statistically significant.

## Electronic supplementary material

supplementary information
