# Supplementary material for: Hydrogen gas protects IP3Rs by reducing disulfide bridges in human keratinocytes under oxidative stress
Source: Sci Rep. 2017 Jun 15;7:3606. doi: 10.1038/s41598-017-03513-2 (PMC5472599; doi:10.1038/s41598-017-03513-2)
Supplement: Supplementary file 1 — supplementary information [file 41598_2017_3513_MOESM1_ESM.pdf]

Supplementary Material for:

Hydrogen gas protects IP3Rs by reducing disulfide bridges in human keratinocytes under oxidative stress

Ching-Ying Wu[¶], Wen-Li Hsu[¶], Ming-Hsien Tsai, Jui-Lin Liang, Jian-He Lu, Chia-Jung Yen, Hsin-Su Yu, Mami Noda, Chi-Yu Lu, Chu-Hhung Chen, Shian-Jang Yan[*], and Tohru Yoshioka[*]

Supplementary Table S1

RPTPα / Sequence	Average normalized abundances (counts)				
	Modifications (Delta mass)	In quantitation	vehicle	H_2O_2	H_2O_2+2-ME
QQQQSGNHPITVHCSAGAGR (Cys-723)	MPB (646.25)	yes	20900	23100	15900

Supplementary Table S1. Proteomics analysis for the cysteine-labeling peptides in RPTPα. LC-MS/MS analyzed MBP-modified peptides sequences with delta mass 646.25 (Cys+MBP) and isotopic quantification. Average normalized abundances reveals each peptide signal counts with three tests. The modified Cys residue is indicated by residue number in RPTPα.
